# Co-infection patterns of vector-borne zoonotic pathogens in owned free-ranging dogs in central Chile

**DOI:** 10.1007/s11259-022-10009-6

**Published:** 2022-11-03

**Authors:** Aitor Cevidanes, Sophia Di Cataldo, Catalina Muñoz-San Martín, Maria Stefania Latrofa, Claudia Hernández, Pedro E. Cattan, Domenico Otranto, Javier Millán

**Affiliations:** 1 Programa de Doctorado en Medicina de la Conservación, Facultad de Ciencias de la Vida, Universidad Andrés Bello, República 440, Santiago, Chile; 2 Department of Animal Health, NEIKER-Basque Institute for Agricultural Research and Development. Basque Research and Technology Alliance (BRTA), Parque Científico y Tecnológico de Bizkaia, P812, 48160 Derio, Spain; 3 Escuela de Medicina Veterinaria, Facultad de Ciencias Médicas, Universidad Bernardo O’Higgins, 8370854 Santiago, Chile; 4 Department of Veterinary Medicine, University of Bari, 70010 Valenzano, Bari Italy; 5 Escuela de Medicina Veterinaria, Facultad de Ciencias de la Vida, Universidad Andres Bello, República 252, Santiago, Chile; 6 Facultad de Ciencias Veterinarias y Pecuarias, Universidad de Chile, Santiago, Región Metropolitana Chile; 7 Facultad de Ciencias de la Vida, Universidad Andres Bello, República 440, Santiago, Chile; 8 Instituto Agroalimentario de Aragón-IA2 (Universidad de Zaragoza-CITA), Miguel Servet 177, 50013 Zaragoza, Spain; 9 Fundación ARAID, Avda. de Ranillas, 50018 Zaragoza, Spain; 10 Instituto de Medicina y Biología Experimental de Cuyo (IMBECU) , Consejo Nacional de Investigaciones Científicas y Tecnológicas (CONICET), Mendoza, Argentina

**Keywords:** *Canis lupus familiaris*, Chagas disease, Flea-borne, Tick-borne, Vector-borne

## Abstract

We investigated the co-occurrence of the nine of the most relevant canine vector-borne pathogens (CVBP) using conventional and real-time PCR and evaluated risk factors and potential non-apparent haematological alterations associated with co-infection in 111 rural, owned, free-ranging dogs in the Metropolitan Region of Chile.

At least one pathogen was detected in 75% of the dogs. DNA of *Anaplasma platys* (Ap; 36%), *Candidatus* Mycoplasma haematoparvum (CMhp; 31%), *Mycoplasma haemocanis* (Mhc; 28%), *Trypanosoma cruzi* (17%), *Leishmania* spp. (4.5%), and *Acanthocheilonema reconditum* (1%) was detected. All dogs were negative for *Ehrlichia* spp., *Rickettsia* spp., *Bartonella* spp., Piroplasmida, and *Hepatozoon* spp. Thirty-eight dogs (34%) were coinfected. CMhp was involved in 71%, Mhc in 58%, and Ap in 50% of the co-infections. The most common co-infection pattern was CMhp–Mhc (37% of the cases). The prevalence of Ap was higher in juvenile than in adult dogs, whereas the opposite was found for CMhp and Mhc. Adult dogs were four times more likely of being co-infected than juveniles. Co-infected animals showed higher white blood cell count, segmented neutrophil count, and GGT levels than non-co-infected dogs. Clinically healthy but infected dogs may act as reservoirs of CVBP, and their free-ranging behavior would facilitate the spread of these pathogens to other dogs as well as human beings or wild carnivores.

**Supplementary Information:**

The online version contains supplementary material available at 10.1007/s11259-022-10009-6.

## Highlights

DNA of at least one of nine vector-borne pathogens found in 75% of rural dogs.

*Anaplasma platys* was most prevalent but *C*. M. haematoparvum was involved in more coinfections.

Adults were four times more likely of being co-infected than juveniles.

Most infections were subclinical, so dogs act as silent reservoirs of pathogens.

**Supplementary Information:**

The online version contains supplementary material available at 10.1007/s11259-022-10009-6.

## Introduction

Canine vector-borne pathogens (CVBP) comprise a relevant and globally distributed group of disease agents (i.e., viruses, bacteria, protozoa, and helminths) transmitted by hematophagous arthropods such as ticks, fleas, lice, triatomines, mosquitoes, and sand flies (Otranto et al. 2009b; Mullen and Durden 2019). The distribution of some vectors and the pathogens they transmit is changing and the transmission risk is increasing due, among other factors, to climate change (Haines et al. 2006; Beugnet and Marié 2009; Colwell et al. 2011). The increased mobility and worldwide distribution of domestic dogs and cats have also contributed to the rapid extension of some vector arthropods and CVBP (Shaw et al. 2001). Furthermore, the importation of dogs from endemic areas has resulted in an overall increased number of diagnoses of canine vector-borne diseases (CVBD) in previously non-endemic areas (Otranto et al. 2009a). In addition to canine welfare, CVBD is attracting a growing medical interest due to the zoonotic nature of some of those pathogens (Otranto et al. 2009b; Irwin 2014). An extended range of clinical manifestations characterizes the outcomes of CVBDs, according to host individual factors, as well as the occurrence of co-infection with more than one agent (De Tommasi et al. 2013). Hematological and biochemical abnormalities induced by CVBP are often unpredictable, especially when the dog has become co-infected by two or more organisms (Otranto et al. 2009c).

Rickettsial bacteria of the genus *Anaplasma*, *Ehrlichia*, and *Rickettsia* have been molecularly detected in dogs and associated ectoparasites in different regions of Chile (Abarca et al. 2007, 2012, 2013; López et al. 2012a; Poo-Muñoz et al. 2016; Cevidanes et al. 2018; Di Cataldo et al. 2021a). Hemotropic *Mycoplasma* spp., also known as hemoplasmas, have been also broadly detected in dogs all across Chile (Soto et al. 2017; Di Cataldo et al. 2020a; Cataldo et al. 2021b). In contrast, the molecular presence of bacteria of the *Bartonella* genus in dogs and their ectoparasites has been less studied (Pérez-Martínez et al. 2009; Cevidanes et al. 2018; Müller et al. 2018). Vector-borne protozoa have not been widely studied in Chilean dogs either. Although Chile is an endemic region for Chagas disease, caused by the parasite *Trypanosoma cruzi*, few studies have been published in the last decades about the molecular presence of this parasite in dogs (Ortiz et al. 2016; Opazo et al. 2021). The only canine Piroplasmida, molecularly confirmed in dogs in Chile is *Babesia vogeli* (Di Cataldo et al. 2020b), but it appears to be restricted to some areas (Di Cataldo et al. 2022). At least three variants of *Hepatozoon* spp. have been described in foxes in the country, but not in dogs (Di Cataldo et al. 2022). DNA and antibodies against *Leishmania* sp. were recently described in Chile (Di Cataldo et al. 2022). Regarding vector-borne filaroids, *Acanthocheilonema* spp. and *Dirofilaria repens* have been detected in dogs in Chile (Alcaíno and Rudolph 1970; Alcaíno et al. 1984; López et al. 2012b; Di Cataldo et al. 2022). *Acanthocheilonema* sp. was also found in an Andean fox (*Lycalopex culpaeus*) from Chile (Oyarzún-Ruiz et al. 2020).

The dog population in Chile was estimated at 4.059.200 individuals (Gompper 2014), and owned free-roaming dogs (i.e. characterized by the lack of continuous direct supervision and irresponsible ownership) are common in Chile (Villatoro et al. 2016). Owned free-ranging dogs are considered the intermediate stage between well-managed pets with movement restrictions and feral dogs without human control and management (Bonacic et al. 2019). In Chile, prophylactic measures such as antiparasitic treatments are infrequently applied to rural dogs by their owners (Poo-Muñoz et al. 2016). This is why these animals are useful sentinels for vector and pathogen environmental pressure in a given area (Cardoso et al. 2012; Dantas-Torres et al. 2012). Free-ranging dog lifestyle is indeed considered an important factor for parasite or pathogen transmission (Otranto et al. 2017). Outdoor and/ or hunting lifestyle has been associated with higher exposure to some CVBP when compared with indoor and pet lifestyles (Solano-Gallego et al. 2006; Checa et al. 2019).

Despite the diversity of studies carried out in Chile detecting CVBP, the concomitant presence of different agents and the impact of being co-infected on dogs’ health have never been evaluated to date. Nevertheless, coinfection is the rule more than the exception (Brooker 2010). The complexity of the so-called ‘host-parasite ecosystems’ includes a variety of direct and indirect interactions between hosts and pathogens. For example, acquired immunity to one pathogen species may have negative effects on a second species, but can also produce immunosuppression, increasing infection susceptibility (Telfer et al. 2008).

Since all the studies in Chile addressed infection in dogs by a single vector-borne pathogen, the actual burden of CVBP has likely been underestimated. This study aimed to determine the presence and co-occurrence of nine of the most relevant CVBP in free-ranging, owned, rural dogs of central Chile, and to evaluate infection risk factors and potential “hidden” haematological alterations associated with the concurrent infection by two or more pathogens.

## Materials and methods

### Study area and dog sampling

**Fig. 1:**
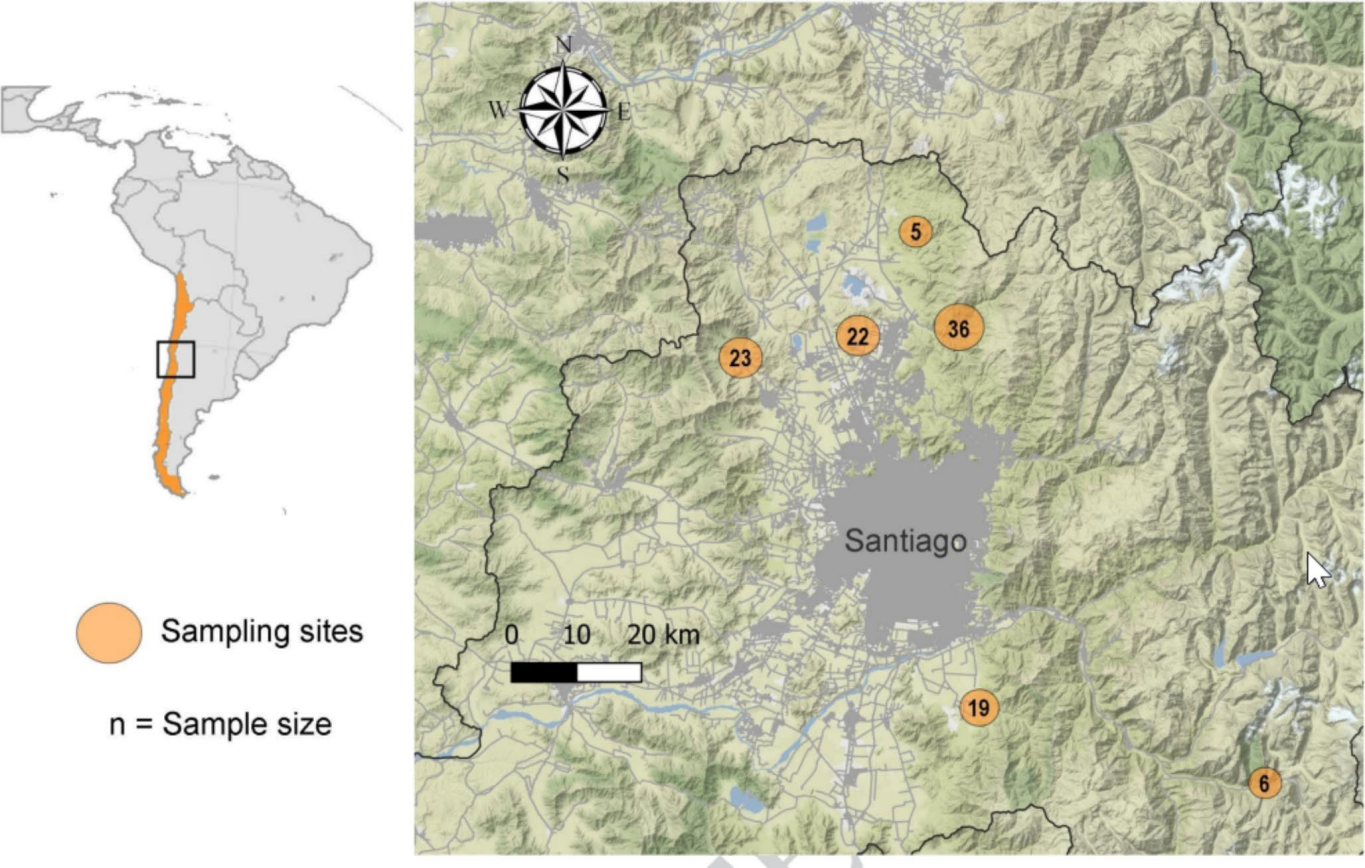
Map of the study area. Circles correspond to dog sampling areas. Numbers indicate the sample size

The study was conducted in the Metropolitan Region of Chile (Fig. 1), which has a typical Mediterranean climate, with a mean annual temperature of around 14.7ºC and annual precipitation of 243.3 mm (INE 2017). From 2016 to 2018, 111 free-roaming rural dogs were sampled and examined in situ. All sampled animals were free-ranging (without permanent confinement). Age estimation (based on tooth eruption) and sex of the dogs were recorded, and a general clinical sign examination was carried out. Dogs were methodically inspected for ectoparasites for 5 min. The data about the prevalence and abundance of ticks and fleas in these dogs were published elsewhere (Cevidanes et al. 2021). Blood obtained from the cephalic vein was collected in two separated EDTA tubes and a further tube with a serum separator. The serum was removed after centrifugation and frozen at -20ºC until biochemistry analysis. Hematological analyses were performed on whole blood and the remaining sample was frozen at -20° until molecular analysis.

### Laboratory analysis

DNA extraction from 100 μl of blood was performed using DNeasy Blood & Tissue Kit (QIAGEN, Hilden, Germany) according to the manufacturer’s instructions. DNA was eluted in 200 µl of elution buffer. An internal control PCR targeting the RPS19 gene for canine genomic DNA was carried out in all samples examined (Brinkhof et al. 2006). Primers and protocols for pathogen DNA detection are presented in Supplementary Table 1. Briefly, DNA of hemotropic *Mycoplasma* spp., *Bartonella* spp., *Rickettsia* spp., Anaplasmataceae (*Anaplasma* spp. and *Ehrlichia* spp.), Piroplasmida (*Babesia* spp. and *Theileria* spp.), and *Hepatozoon* spp. was screened by conventional PCR (cPCR) with the primers and run protocols previously described (Millán et al. 2019). The prevalence of three of the pathogens was included in country-wide surveys published elsewhere (Di Cataldo et al. 2021a; Cataldo et al. 2021b, 2022). Samples scored positive for *Mycoplasma* were examined with specific primers for *Mycoplasma haemocanis* (Mhc) and *Candidatus* Mycoplasma haematoparvum (CMhp) to detect coinfections (Watanabe et al. 2008; Martínez-Díaz et al. 2013). *Trypanosoma cruzi* was detected and quantified by real-time PCR following the protocols described by Yefi-Quinteros et al. (2018). *Leishmania* spp. DNA was screened by conventional PCR using the protocol described by Cortés et al. (2004)  and positive samples were further analyzed by qPCR using primers and run protocol previously described by Francino et al. 2006) 2004 for sequencing purposes. Filaroids were screened by cPCR as described by Casiraghi et al. (2001). To avoid cross-contamination, DNA extraction, mixing of DNA-free PCR reagents, and the addition of the template DNA was carried out in separate areas with separate equipment and solutions. PCR products were visualized on a 2% agarose electrophoresis gel and later purified and sequenced by the Sanger technique. Obtained sequences were then compared with those available in GenBank® by BLAST analyses (http://www.ncbi.nlm.nih.gov/blast).

### Hematology and serum chemistry

The following hematological parameters were analyzed through manual and automatic cell counter (HumaCount 80TS©, Human, Germany): hematocrit (HCT), red blood cell (RBC), platelet (PLT) and total leukocyte count (WBC), hemoglobin concentration (HGB), mean corpuscular volume (MCV) and mean corpuscular hemoglobin concentration (MCHC). Relative leukocyte differentiation was performed by microscopic observation. The following serum biochemistry parameters were evaluated using Analyzer BA400© (BioSystems, Spain): total proteins, albumin, calcium, phosphorus, cholesterol, glucose, creatinine, urea, blood urea nitrogen (BUN), aspartate aminotransferase (AST), alanine transaminase (ALT), alkaline phosphatase (ALP) and gamma-glutamyl transferase (GGT).

### Data analysis

Confidence intervals for prevalence were calculated using the “EpiR” package of R software. Parasitological terms follow Bush et al. (1997). Differences in the occurrence of pathogens, the existence of coinfection, and the number of pathogens per host depending on the dog’s sex (male/female) and age (young/adult) were evaluated. For *Anaplasma platys*, Mhc, and CMhp, the prevalence, and abundance of *Rhipicephalus sanguineus* sensu stricto were also analyzed as independent variables. Generalized linear mixed models (GLMMs) were used to study the binary variables (i.e., pathogen occurrence = absence/presence; pathogen coinfection = not coinfected/ coinfected) and fixed and random effects. GLMMs handle non-normal data by using link functions and exponential family distributions and incorporate random effects (Bolker et al. 2009). The study zone (Andean hillside, central valley, and coastal hillside) was included as a random effect. GLMMs were analyzed using the “lme4” package of R software with binomial error (logit-link function). The best model was selected using the “dredge” function from the “MuMIn” package, which generates, given a full model, a subset of models and selects the best model that best fits the data, based on Akaike information criterion corrected to sample size (AICc). The overall fit of the best model was assessed by residuals analysis and comparison with the null model (with an intercept and random effects only), using the likelihood ratio test. Individuals with information on all factors were included in the models. In the case that a category of the independent variables had not any positive animal, the evaluation of that variable was carried out by Fisher’s exact test. In that case, that variable was removed from the full model of GLMM analysis. Differences in hematological and biochemistry values were tested using Student’s *t* or Mann Whitney U depending on data distribution. Initially, differences between adult (older than one year) and young dogs (younger than one year) were evaluated. In case of not finding significant differences between ages, these were pooled to assess the association between parameter and co-infection status and otherwise were analyzed separately. All statistical analyses were carried out using R software.

## Results

### Pathogen occurrence and co-infection patterns

**Fig. 2:**
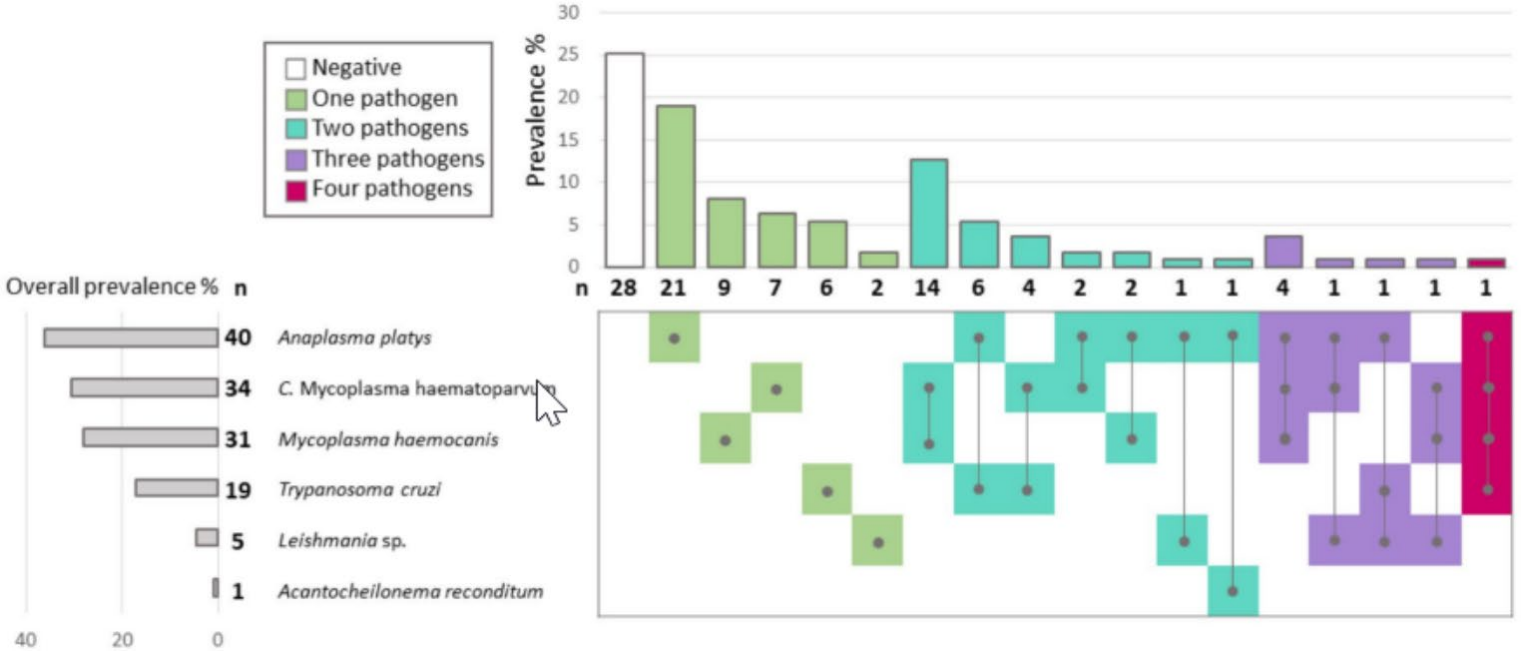
Number of positive animals and observed prevalence for each pathogen and each co-infection pattern in rural dogs sampled in central Chile

Seventy-five percent of the dogs were infected with at least one CVBP (Table 1). Anaplasmataceae DNA was found in 40 dogs (36%; Table 1, Fig. 2) and sequencing confirmed that all amplicons corresponded to *A. platys*. Hemoplasmal DNA was found in 45 dogs, for an overall prevalence of 40.5% (95% Confidence Intervals = 31.4–49.7). CMhp and Mhc DNA were confirmed, respectively, in 34 (30%) and 31 (28%) dogs. DNA of *T. cruzi* was detected in 19 dogs (17%), with a parasite load of one *T. cruzi* parasite equivalent/mL. Using both qPCR and cPCR methods, we found samples that scored positive for *Leishmania* spp. in five dogs (4.5%). Unfortunately, no readable sequences were obtained. One dog was positive for filariae, and the obtained sequence showed 99.4% identity with an *A. reconditum* available in GenBank (JF461456.1). All dogs were negative for *Rickettsia* spp., *Bartonella* spp., Piroplasmida, and *Hepatozoon* spp. Thirty-eight dogs (34%) were infected with more than one pathogen (Table 1, Fig. 2). Among them, 30 animals were infected by two pathogens, seven by three pathogens, and one by four pathogens. The most common co-infection pattern was CMhp – Mhc (n = 14/38, 36.8%). CMhp was involved in 71.0% of the co-infections (n = 27), Mhc in 57.8% (n = 22) and *A. platys* in 50% of them (n = 19).

**Table 1:** Occurrence of selected canine vector-borne pathogens in rural dogs in Chile and co-infection depending on host sex and age, and the mean abundance of *Rhipicephalus sanguineus* for tick-borne pathogens. All animals were negative for *Rickettsia* spp., *Bartonella* spp., Piroplasmida, and *Hepatozoon* spp

		*Anaplasma platys*		*C.*Mycoplasma haematoparvum		*Mycoplasma haemocanis*		*Trypanosoma cruzi*		*Leishmania* sp.		*Acanthocheilonema reconditum*		Co-infection
		% (95%CI)		% (95%CI)		% (95%CI)		% (95%CI)		% (95%CI)		% (95%CI)		% (95%CI)
Overall prevalence		36.0 (27.1–44.9)		30.6 (22.0-39.2)		27.9 (19.6–36.3)		17.1 (10.1–24.1)		4.5 (0.6–8.4)		0.9 (0.0-2.6)		34.2 (25.4–41.1)
Sex														
Female		30.9 (16.9–44.9)		23.8 (10.9–36.6)		21.4 (9.0-33.8)		28.6 (14.9–42.2)		9.5 (0.6–18.4)		0		38.1 (23.4–52.7)
Male		39.1 (27.6–50.6)		34.8 (23.5–46.0)		31.9 (20.8–42.8)		10.1 (3.0-17.2)		1.4 (0.0-4.3)		1.4 (0.0-4.3)		33.3 (22.2–44.4)
Age														
Adult		28.7 (19.2–38.2)*		39.1 (28.8–49.3)*		33.3 (23.4–43.2)*		17.2 (9.3–25.1)		4.6 (0.2-9.0)		1.1 (0.0-3.3)		41.4 (31.0-51.7)*
Juvenile		62.5 (43.1–81.9)*		0*		8.3 (0.0-19.4)*		16.7 (1.7–31.5)		4.2 (0.0-12.2)		0		12.5 (0-25.7)*
*R.s.* MA		MA ± SE		MA ± SE		MA ± SE								
Infected		7.2 ± 2.6*		5.3 ± 1.4		7.0 ± 2.9		-		-		-		-
No-infected		3.0 ± 0.6*		4.1 ± 1.3		3.5 ± 0.8		-		-		-		-

95%CI: 95% Confidence Intervals; * significant differences between groups; *R.s*.; “*Rhipicephalus sanguineus S.S.*”* s.s.ss*; MA, mean abundance; SE, standard error

### Risk factor analysis

**Fig. 3:**
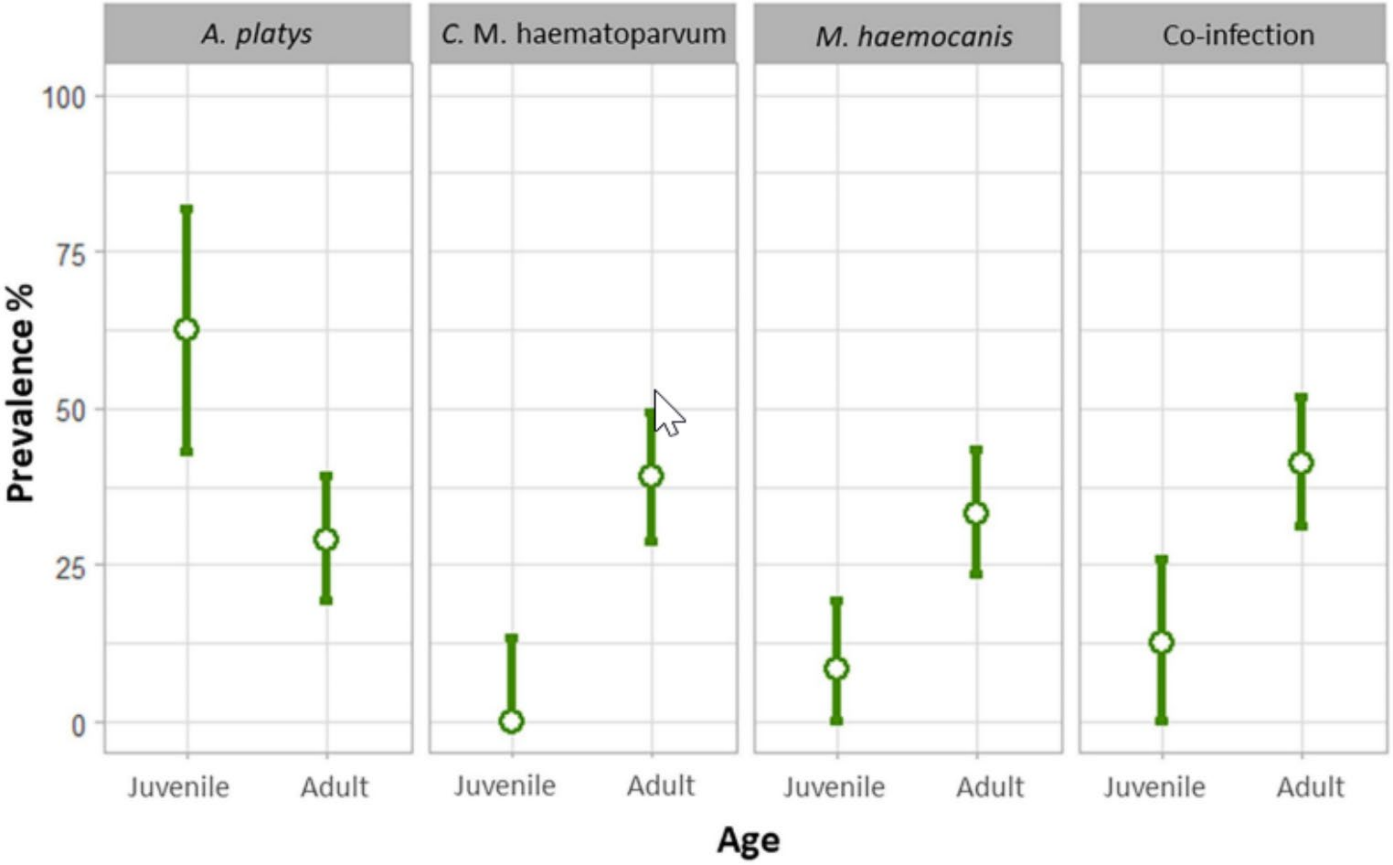
Differences in prevalence of *Anaplasma platys*, *Candidatus* Mycoplasma haematoparvum, *Mycoplasma haemocanis*, and co-infection (these pathogens plus *Trypanosoma cruzi*, *Leishmania* sp., and/or *Acanthocheilonema reconditum*) depending on the age class of rural dogs sampled in central Chile. All these differences were statistically significant

**Fig. 4:**
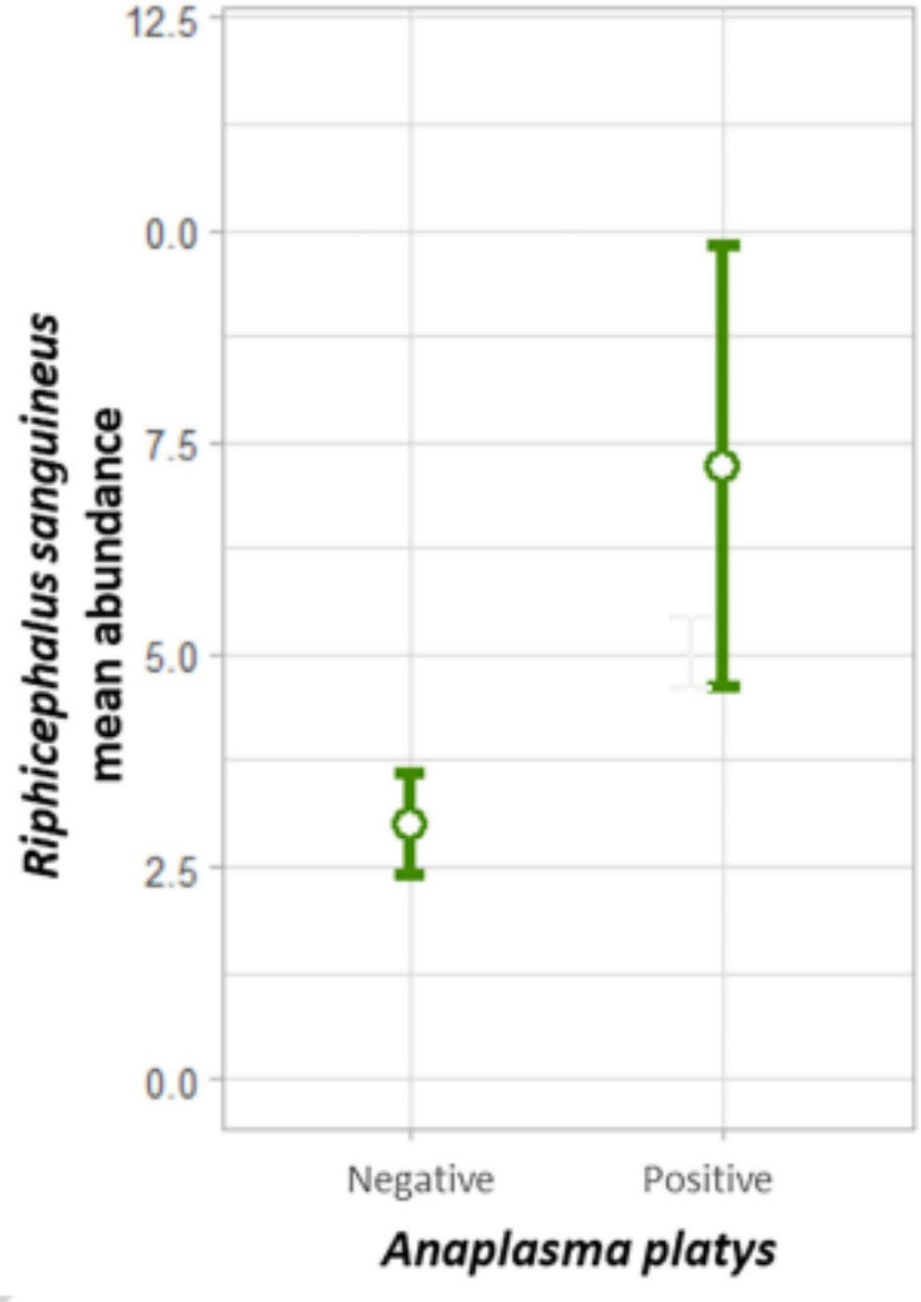
Abundance of *Rhipicephalus sanguineus* sensu stricto depending on the *Anaplasma platys* infection status

The probability of being infected by *A. platys* was four times higher (OR = 4.13, 95%CI = 1.60-10.66; z-value = 2.93; p = 0.003) for a juvenile than for an adult dog (Table 1, Fig. 3). In contrast, adult age was associated with a higher prevalence for CMhp (Fisher’s p = 0.0001) and Mhc (OR = 5.49, 95%CI = 1.2-25.01, z-value = 2.51, p = 0.01). Dogs infected by *A. platys* showed a higher abundance of *R. sanguineus* than those non-infected (z-value = 1.947, p = 0.05; Table 1, Fig. 4). The prevalence and abundance of *R. sanguineus* were not related to the presence of any other agent. Adult dogs were five times more likely of being co-infected than juveniles (OR = 4.9, 95%CI = 1.4–17.8, z-value = 2.44, p = 0.01) (Fig. 3).

### Clinical, hematological, and biochemical findings

**Fig. 5:**
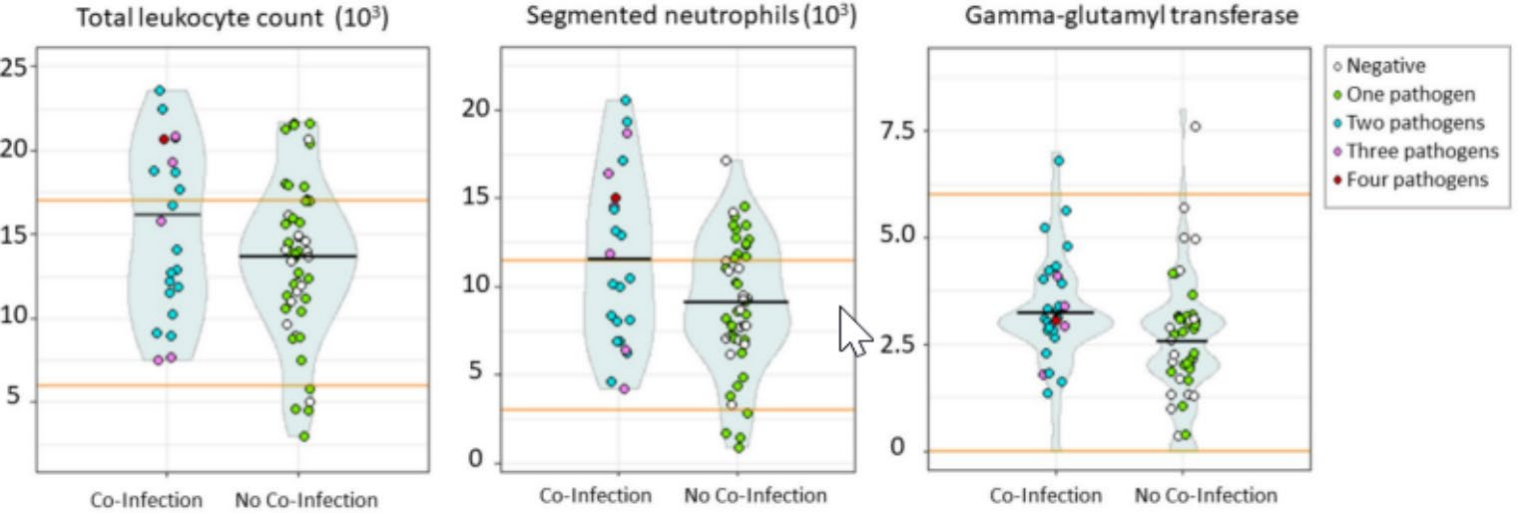
Differences in total leukocyte count, segmented neutrophil count, and gamma-glutamyl transferase (GGT) depending on the co-infection status. All these differences were statistically significant. Black lines indicate the mean and orange lines the maximum and minimum reference values based on Thrall et al. (2012)

Most of the animals were considered apparently healthy in the physical evaluation. Only eight of the dogs (7.2%) presented pale mucous membranes, without differences between co-infected and non-co-infected animals (Fisher’s p = 1). Co-infected animals showed significant higher white blood cell count (WBC) (t = 2.01, p < 0.05) and segmented neutrophil count (t = 2.46, p < 0.05) and GGT levels (U = 583.5, p < 0.05) (Fig. 5; Supplementary Table 2).

## Discussion

The present study is the most extensive study ever conducted in the most relevant CVBP in Chile. We documented frequent rates of infection (inferred from DNA detection) in these dogs, with up to three-quarters of the individuals positive for at least one pathogen. The outdoor activity of the studied free-ranging dogs exposes them to a range of vectors. Although we did not collect information in this regard about the sampled dogs, rural dogs in Chile are rarely subjected to antiparasitic prophylactic treatments. Previous studies in other parts of the world showed that rural dogs are frequently exposed to or infected by different vector-borne pathogens (Proboste et al. 2015; Dantas-Torres et al. 2018), and higher rates of exposure or infection were found in rural dogs when compared with their urban counterparts (Lim et al. 2010; Vieira et al. 2012; Costa-Júnior et al. 2013). In the Metropolitan Region of Chile, the prevalence of *R. sanguineus* and *Ctenocephalides canis* was indeed higher in rural than in urban dogs (Abarca et al. 2016).

*Anaplasma platys* was the only Anaplasmataceae confirmed in this study, as for the whole country (Di Cataldo et al. 2021a). This picture is similar to that reported in other geographical areas where the temperate lineage of *R. sanguineus* s.s. is the only tick species infecting dogs (Latrofa et al. 2014; Otranto et al. 2019). The higher prevalence of *A. platys* infections in juvenile dogs in our study was already been recorded in a previous study in Africa (Matei et al. 2016), most likely due to a primary exposure of young individuals to the pathogen (Otranto et al. 2010; de Caprariis et al. 2011) and might be related to the lower levels CD8 T lymphocytes found in young dogs (Greeley et al. 1996), which have a role in the clearance of rickettsial infections (Walker et al. 2015). Overall, our results suggest that the risk of infection with *A. platys* is more associated with the abundance of the tick than just the presence of the tick. In agreement with our results, other studies found that dogs infested with *R. sanguineus* were more likely to be infected with or exposed to *Anaplasma* spp. than uninfested dogs (Costa-Júnior et al. 2013; Rojas et al. 2014; Piantedosi et al. 2017; Di Cataldo et al. 2021a).

Hemoplasmas were the second more abundant CVBP detected in this survey. Rural environments and free-ranging behavior were pointed out as risk factors for hemoplasma infections (Biondo et al. 2009; Soto et al. 2017, Aktas and Ozubek 2018). Interestingly, the prevalence of the two hemoplasma species in the studied dog population differs when co-infections are evaluated. When comparing the prevalence obtained with our specific primers with the screening protocol and direct sequencing alone, as reported by Di Cataldo et al. (2021b), prevalence increased from 21% to 28% for Mhc and from 13.5% to 31% for CMhp. The reason for this difference could be due to a lower bacteremia level of CMhp than of Mhc and must be taken into account when studying these pathogens. On the other hand, the observed higher infection percentage in older dogs may be explained by an increased probability of exposure throughout life and/or by the characteristic long-term bacteremia of hemoplasma infection (Willi et al. 2010; Greene 2013). In this sense, a lack of hemoplasma clearance was reported in infection follow-up studies (Wengi et al. 2008; Hulme-Moir et al. 2010).

To the best of our knowledge, this survey represents the second molecular detection of *T. cruzi* in dogs in central Chile (Opazo et al. 2021), although the presence of parasitized dogs in this region was known in the past (Schenone et al. 1991). Dogs are competent hosts with importance in the cycle of *T. cruzi* in endemic areas (Esch and Petersen 2013), being signaled as a bridge between the domestic and sylvatic transmission cycles (Ramírez et al. 2013). This can be the case in our study area, where all of the studied dogs live outdoor and some of them accompany mule drivers in areas where triatomines abound (Cattan et al. 2002). Further studies should aim to characterize the genetic diversity of *T. cruzi* in the region.

A third of the studied dogs were co-infected with two or more pathogens. Co-infection is considered frequent in CVBD-endemic areas, especially in dogs living in environments with high vector density and without antiparasitic treatment (Otranto et al. 2009c). Interestingly, although *A. platys* was the most prevalent agent in our study, was not the pathogen most commonly associated with co-infection in dogs, in contrast with previous studies carried out in areas where *R. sanguineus* is prevalent (Otranto et al. 2010). In our case, hemoplasma species were common in cases of co-infection, and concomitant infections have indeed been considered a risk factor for hemoplasma infection (Roura et al. 2010; Aktas and Ozubek 2018).

Higher WBC and segmented neutrophil levels were found in co-infected animals. No consistent leukogram abnormalities have been associated with canine hemoplasmosis or anaplasmosis (Greene 2013; Sainz et al. 2015; Soto et al. 2017). However, increased leukogram values have been associated with *T. cruzi* infections (Villalba-Alemán et al. 2019). On the other hand, higher GGT values were found in co-infected animals. Anyway, almost all the GGT values were in the range of the reference values (Thrall et al. 2012). Our findings may be explained by the absence of acute stages of infection. Chronically infected dogs usually present low bacteremia or parasitemia (Otranto et al. 2009c). Thus, dogs with chronic or “hidden” infections used to be healthy with absent or minor hematological abnormalities (Otranto et al. 2009c; de Caprariis et al. 2011). For example, most of the cases of canine hemoplasmosis used to be chronic subclinical infections and infected dogs seemed unable to clear the infection (Willi et al. 2010). Therefore, as suggested before, co-infection complicates the diagnosis based on clinical examination and hematological and biochemistry abnormalities alone (Otranto et al. 2009c). Moreover, it has to be mentioned that many other parasites (helminths) and pathogens (viruses, bacteria) probably infecting the studied dogs were not tested. It has been shown that neglecting some taxa of the host-parasite community diminishes the chances of detecting the cost of infection (Serrano and Millán 2014).

## Conclusion

Rural, owned free-ranging dogs of central Chile are infected or parasitized by a range of agents of veterinary and potentially zoonotic interest. It is important to remark that those clinically healthy but infected dogs could be acting as subclinical carriers of different CVBP, possibly contributing to the spreading of some of these pathogens to potential vectors and among their owners, other dogs, or protected wild carnivores. Their free-ranging behavior would further facilitate their role as uncontrolled reservoirs and a bridge between anthropized and natural environments. In consequence, we believe that authorities must promote among dog owners in rural areas of Chile the use of prophylactic measures, such as the periodic application of antiparasitic products to diminish the burden of ticks, fleas, and vector-borne pathogens. Dogs should not be allowed to roam free and their confinement in the household should be enforced.

## Electronic supplementary material

Below is the link to the electronic supplementary material.

Supplementary Material 1

Supplementary Material 2

## Data Availability

The datasets generated during and/or analysed during the current study are available from the corresponding author on reasonable request.
