# Supplementary material for: Co-infection patterns of vector-borne zoonotic pathogens in owned free-ranging dogs in central Chile
Source: Vet Res Commun. 2022 Nov 3;47(2):575–85. doi: 10.1007/s11259-022-10009-6 (PMC10209262; doi:10.1007/s11259-022-10009-6)
Supplement: Supplementary file 1 — Supplementary Material 1 [file 11259_2022_10009_MOESM1_ESM.docx]

**Supplementary file 2**. Hematological and serum chemistry profiles of rural dogs in central Chile depending on the coinfection status.

|  |  | No Co-infected | | | | | |  | Co-infected | | | | | |
| --- | --- | --- | --- | --- | --- | --- | --- | --- | --- | --- | --- | --- | --- | --- |
| Parameter |  | N | Mean | SD | Median | Min | Max |  | N | Mean | SD | Median | Min | Max |
| RBC (10^6^/mm^3)^ |  | 48 | 6.6 | 1.2 | 6.6 | 4.3 | 9.5 |  | 24 | 6.59 | 1.8 | 6.9 | 3.8 | 9.1 |
| Hemoglobine (g/dl) |  | 48 | 14.3 | 3.39 | 14.5 | 3.3 | 21.4 |  | 24 | 14.6 | 2.9 | 15.6 | 7.1 | 18.2 |
| Hematocrite (%) |  | 48 | 45.4 | 10.76 | 44.4 | 12.1 | 64,8 |  | 24 | 47.2 | 8.7 | 50.1 | 25.3 | 58.7 |
| MCV |  | 48 | 68.3 | 6.3 | 70 | 38.4 | 79 |  | 24 | 69.5 | 4.2 | 70 | 59 | 77 |
| MCHC |  | 48 | 31.33 | 2.91 | 31.20 | 18 | 38.6 |  | 24 | 30.8 | 1.2 | 30.8 | 27.8 | 33.8 |
| Platelets (10^3^/ mm^3^) |  | 43 | 179.04 | 132.32 | 191.0 | 4.8 | 451.0 |  | 24 | 209.67 | 156.29 | 183.50 | 12.0 | 518.0 |
| WBC (10^3^/ mm^3^)* |  | 48 | 13.66 | 4.45 | 13.90 | 4.48 | 21.6 |  | 24 | 16.15 | 5.79 | 16.25 | 7.51 | 26.90 |
| Lymphocytes (mm^3^) |  | 49 | 3989 | 2131 | 3498 | 1212 | 14873 |  | 24 | 3833 | 1354 | 3852 | 1115 | 8100 |
| Monocytes (mm^3^) |  | 49 | 840 | 1092 | 581 | 65 | 6840 |  | 24 | 756 | 603 | 559 | 97 | 2252 |
| Seg. neutrophils (mm^3^)* |  | 48 | 9088 | 3588 | 8927 | 855 | 17135 |  | 24 | 11566 | 4773 | 11141 | 4220 | 20578 |
|  |  |  |  |  |  |  |  |  |  |  |  |  |  |  |
| Total proteins |  | 39 | 6.13 | 1.33 | 6.35 | 2.31 | 8.81 |  | 29 | 6.48 | 0.81 | 6.46 | 4.27 | 8.37 |
| Albumin |  | 49 | 2.58 | 0.76 | 2.8 | 0.1 | 3.6 |  | 29 | 2.57 | 0.49 | 2.6 | 1.4 | 3.3 |
| Calcium |  | 45 | 7.40 | 1.78 | 7.79 | 1.98 | 10.34 |  | 29 | 7.62 | 1.44 | 7.97 | 3.59 | 9.99 |
| Phosphorus |  | 39 | 4.88 | 1.42 | 4.6 | 3 | 11.2 |  | 29 | 4.57 | 0.83 | 4.5 | 2.8 | 6.4 |
| Cholesterol |  | 46 | 179.65 | 70.07 | 170.5 | 55 | 388 |  | 29 | 176.34 | 64.48 | 179 | 62 | 275 |
| Glucose |  | 39 | 64.33 | 20.03 | 58 | 38 | 147 |  | 29 | 59.65 | 19.93 | 56 | 37 | 121 |
| Crea |  | 43 | 1.13 | 0.39 | 1.12 | 0.35 | 1.82 |  | 29 | 1.10 | 0.42 | 1.08 | 0.08 | 2.77 |
| Urea |  | 38 | 32.10 | 17.10 | 28.5 | 6.13 | 86 |  | 29 | 37.44 | 12.92 | 35 | 17 | 75 |
| BUN |  | 38 | 15.28 | 7.74 | 13.80 | 4.65 | 40.29 |  | 29 | 17.46 | 6.03 | 16.51 | 7.81 | 35.03 |
| AST |  | 42 | 29.85 | 16.79 | 28 | 7 | 89 |  | 29 | 30.55 | 16.81 | 30 | 9 | 98 |
| ALT |  | 42 | 33.61 | 22.80 | 28.5 | 8 | 114 |  | 29 | 34 | 16.28 | 31 | 12 | 89 |
| ALP |  | 44 | 29.36 | 19.34 | 26.5 | 0 | 93 |  | 29 | 23.93 | 11.13 | 20 | 5 | 44 |
| GGT* |  | 47 | 2.55 | 1.58 | 3 | 0 | 8 |  | 29 | 3.24 | 1.37 | 3 | 0 | 7 |
| Total bilirubin |  | 48 | 0.10 | 0.04 | 0.1 | 0.02 | 0.21 |  | 29 | 0.09 | 0.03 | 0.09 | 0.04 | 0.16 |

N, number of samples analyzed; SD, standard deviation; Min, minimum value; Max, maximum value; RBC, red blood cell; MCV, mean corpuscular volume; MCHC, mean corpuscular hemoglobin concentration; WBC, total leukocyte count; Seg. Neutrophils, segmented neutrophils; BUN, blood urea nitrogen; AST, aspartate aminotransferase; ALT, alanine transaminase; ALP, alkaline phosphatase; GGT, gamma-glutamyl transferase; *, significant differences between groups.
