# Supplementary material for: Co-infection patterns of vector-borne zoonotic pathogens in owned free-ranging dogs in central Chile
Source: Vet Res Commun. 2022 Nov 3;47(2):575–85. doi: 10.1007/s11259-022-10009-6 (PMC10209262; doi:10.1007/s11259-022-10009-6)
Supplement: Supplementary file 2 — Supplementary Material 2 [file 11259_2022_10009_MOESM2_ESM.docx]

**Supplementary Table 1.** Targeted genes, primers and conditions used for the molecular detection of vector-borne pathogens.

| **Organism** | **Region**  **amplified** | **PCR Type** | **Fragment**  **length (bp)** | **Name (Sense): Nucleotide sequence (5′-3′)** | **Reference** | **PCR condition adaptation** |  |
| --- | --- | --- | --- | --- | --- | --- | --- |
|  |  |  |  |  |  |  |  |
| Canine Internal Control | *RSP19* | Si | 95 | RSP19-F (F): CCTTCCTCAAAAA/GTCTGGG | Brinkhof et al. 2006 | 95°C 5m, 40c (95°C 20s, 55°C 30s, 72°C 15s), 72°C 7m |  |
|  |  |  |  | RSP19-R (R): GTTCTCATCGTAGGGAGCAAG |  |  |  |
|  |  |  |  |  |  |  |  |
| *Mycoplasma* spp. | *16SrRNA* | Si | 384 | Mycop16S rRNA-F (F): ATGTTGCTTAATTCGATAATACACGAAA | Cabello et al. 2013 | 95°C 4m, 40c (95°C 15s, 60°C 30s, 72°C 30s), 72°C 7m |  |
|  |  |  |  | Mycop16S rRNA-R (R): ACRGGATTACTAGTGATTCCAACTTCAA |  |  |  |
|  |  |  |  |  |  |  |  |
| *M. haemocanis* | *16SrRNA* | Si | 175 | Mhf-OH-OK1 (F): ATGCCCCTCTGTGGGGGATAGCCG | Watanabe et al. 2008 | 95°C 5m, 35c (95°C 45s, 58.4°C 45s, 72°C 30s), 72°C 10m |  |
|  |  |  |  | Mycop-00CB-r1 (R): ATGGTATTGCTCCATCAGACTTTCG |  |  |  |
|  |  |  |  |  |  |  |  |
| *Candidatus* M. haematoparvum | *16SrRNA* | Si | 175 | M sp (F): GGAATCACTAGTAATCCYGTGTCAGCTATAT | Martínez-Díaz et al., 2013 | 95°C 4m, 45c (95°C 30s, 56.5°C 30s, 72°C 20s), 72°C 7m |  |
|  |  |  |  | C Mhp (R): AATTAAATACGGTTTCAACTAGTACGTTTCTTT |  |  |  |
|  |  |  |  |  |  |  |  |
| *Bartonella* spp. | *ITS* | Si | 150-240 | BartITSf (F): AGATGATGATCCCAAGCCTTCTG | Cevidanes et al. 2017 | 95°C 10m, 40c (95°C 15s, 62°C 1m, 72°C 30s), 72°C 10m |  |
|  |  |  |  | BartITSr (R): CCTCCGACCTCACGCTTATCA |  |  |  |
|  |  |  |  |  |  |  |  |
| Anaplasmataceae | *16SrRNA* | Si | 345 | EHR16SD (F): GGTACCYACAGAAGAAGTCC | Parola et al. 2000 | 95°C 5m, 40c (95°C 30s, 62°C 30s, 72°C 45s), 72°C 7m |  |
|  |  |  |  | EHR16SR (R): TAGCACTCATCGTTTACAGC |  |  |  |
|  |  |  |  |  |  |  |  |
| *Rickettsia* spp. | *gltA* | Ne | 381 | RpCS.877p (F): GGGGGCCTGCTCACGGCGG | Choi et al. 2005 | 95°C 5m, 35c (95°C 15s, 54°C 15s, 72°C 30s), 72°C 7m |  |
|  |  |  |  | RpCS.1258n (R): TTGCAAAAAGTACAGTGAACA |  |  |  |
|  |  |  | 337 | RpCS.896p (F): GGCTAATGAAGCAGTGATAA |  |  |  |
|  |  |  |  | RpCS.1233n (R): GCGACGGTATACCCATAGC |  |  |  |
| Piroplasmida | *18SrRNA* | Si | 500 | BAB 143-167 (F): CCGTGCTAATTGTAGGGCTAATACA | Soares et al. 2015 | 95°C 5m, 35c (95°C 30s, 58°C 30s, 72°C 40s), 72°C 7m |  |
|  |  |  |  | BAB 694-667 (R): GCTTGAAACACTCTARTTTTCTCAAAG |  |  |  |
|  |  |  |  |  |  |  |  |
| *Hepatozoon* spp. | *18SrRNA* | Si | 670 | HEP 1 mod-F (F): CGCGAAATTACCCAATTCTA | Spolidorio et al. 2009 | 95°C 3m, 40c (95°C 15s, 55°C 40s, 72°C 40s), 72°C 5m |  |
|  |  |  |  | HEP 4-R (R): TAAGGTGCTGAAGGAGTCGTTTAT |  |  |  |
|  |  |  |  |  |  |  |  |
| *Trypanosoma cruzi* | nuclear satellite DNA | q | 166 | Cruzi 1 (F): ASTCGGCTGATCGTTTTCGA  Cruzi 2 (R): AATTCCTCCAAGCAGCGGATA  Dye: EvaGreen® | Yefi-Quinteros *et al.* 2018 | 95°C 15m, 40c (95°C 15s, 65°C 20s, 72°C 20s) |  |
|  |  |  |  |  |  |  |  |
| *Leishmania* spp. | Kinetoplast minicircle DNA | q | 120 | LEISH-1 (F): AACTTTTCTGGTCCTCCGGGTAG  LEISH-2 (R): ACCCCCAGTTTCCCGCC  Probe: AAAAATGGGTGCAGAAAT | Francino et al. 2006 | 95°C 3m, 42c (95°C 10s, 60°C 30s) |  |
|  |  |  |  |  |  |  |  |
| *Leishmani donovani s.l.* | Kinetoplast minicircle DNA | Si | 447 | MC1 (F): GTTAGCCGATGGTGGTCTTG  MC2 (R): CACCCATTTTTCCGATTTTG | Cortes et al. 2004 | 94°C 10m, 35c (94°C 30s, 60°C 20s, 72°C 30s), 72°C 5m |  |
|  |  |  |  |  |  |  |  |
| Filaroids | *cox*1 | Si | 650 | NTF (F): TGATTGGTGGTTTTGGTAA  NTR (R): ATAAGTACGAGTATCAATATC | Casiraghi et al. 2001 | 95°C 10m, 40c (95°C 1m, 52°C 1m, 72°C 1m) 72°C 7m |  |

Si, single conventional PCR; Ne, nested conventional PCR; q, qPCR; s, seconds; m, minutes; c, cycles

**References**

Brinkhof, B., Spee, B., Rothuizen, J., & Penning, L. C. (2006). Development and evaluation of canine reference genes for accurate quantification of gene expression. Analytical Biochemistry, 356(1), 36-43.

Cabello, J., Altet, L., Napolitano, C., Sastre, N., Hidalgo, E., Dávila, J. A., & Millán, J. (2013). Survey of infectious agents in the endangered Darwin's fox (*Lycalopex fulvipes*): High prevalence and diversity of hemotrophic mycoplasmas. Veterinary Microbiology, 167(3-4), 448-454.

Casiraghi, M., Anderson, T.J., Bandi, C., Bazzocchi, C., Genchi, C. (2001). A phylogenetic analysis of filarial nematodes: comparison with the phylogeny of *Wolbachia* endosymbionts. Parasitology, 122, 93-103.

Cevidanes, A., Altet, L., Chirife, A. D., Proboste, T., & Millán, J. (2017). Drivers of *Bartonella* infection in micromammals and their fleas in a Mediterranean peri-urban area. Veterinary Microbiology, 203, 181-188.

Choi, Y. J., Jang, W. J., Kim, J. H., Ryu, J. S., Lee, S. H., Park, K. H., ... & Kim, I. S. (2005). Spotted fever group and typhus group rickettsioses in humans, South Korea. Emerging Infectious Diseases, 11(2), 237.

Cortes, S., Rolão, N., Ramada, J., Campino, L., (2004). PCR as a rapid and sensitive tool in the diagnosis of human and canine leishmaniasis using *Leishmania donovani* s.l. - Specific kinetoplastid primers. Transactions of The Royal Society of Tropical Medicine and Hygiene. 98, 12–17.

Francino, O., Altet, L., Sánchez-Robert, E., Rodriguez, A., Solano-Gallego, L., Alberola, J., Ferrer, L., Sánchez, A., Roura, X., (2006). Advantages of real-time PCR assay for diagnosis and monitoring of canine leishmaniosis. Veterinary Parasitology. 137, 214–221.

Liu H. & Beckenbach A. T. (1992). Evolution of the mitochondrial cytochrome oxidase II gene among 10 orders of insects. Molecular Phylogenetics and Evolution 1, 41 – 52 .

Lv, J., Wu, S., Zhang, Y., Zhang, T., Feng, C., Jia, G., & Lin, X. (2014). Development of a DNA barcoding system for the Ixodida (Acari: Ixodida). Mitochondrial DNA, 25(2), 142-149.

Martínez-Díaz, V.L., Silvestre-Ferreira, A.C., Vilhena, H., Pastor, J., Francino, O., Altet, L., (2013). Prevalence and co-infection of haemotropic mycoplasmas in Portuguese cats by real-time polymerase chain reaction. J. Feline Med. Surg. 15, 879–885

Parola, P., Roux, V., Camicas, J. L., Baradji, I., Brouqui, P., & Raoult, D. (2000). Detection of Ehrlichiae in African ticks by polymerase chain reaction. Transactions of the Royal Society of Tropical Medicine and Hygiene, 94(6), 707-708.

Roux, V., Fournier, P. E., & Raoult, D. (1996). Differentiation of spotted fever group rickettsiae by sequencing and analysis of restriction fragment length polymorphism of PCR-amplified DNA of the gene encoding the protein rOmpA. Journal of Clinical Microbiology, 34(9), 2058-2065.

Roux, V., & Raoult, D. (2000). Phylogenetic analysis of members of the genus *Rickettsia* using the gene encoding the outer-membrane protein rOmpB (ompB). International Journal of Systematic and Evolutionary Microbiology, 50(4), 1449-1455.

Soares, J. F., Carvalho, L., Maya, L., Dutra, F., Venzal, J. M., & Labruna, M. B. (2015). Molecular detection of *Rangelia vitalii* in domestic dogs from Uruguay. Veterinary Parasitology, 210(1-2), 98-101.

Spolidorio, M. G., Labruna, M. B., Zago, A. M., Donatele, D. M., Caliari, K. M., & Yoshinari, N. H. (2009). *Hepatozoon canis* infecting dogs in the State of Espírito Santo, southeastern Brazil. Veterinary Parasitology, 163(4), 357-361.

Watanabe, M., Hisasue, M., Souma, T., Ohshiro, S., Yamada, T., Tsuchiya, R., (2008). Molecular detection of *Mycoplasma haemofelis* and “*Candidatus* Mycoplasma haemominutum” infection in cats by direct PCR using whole blood without DNA extraction. The Journal of Veterinary Medical Science. 70, 1095–1099.

Yefi-Quinteros, E., Muñoz-San Martín, C., Bacigalupo, A., Correa, J.P., Cattan, P.E., (2018). *Trypanosoma cruzi* load in synanthropic rodents from rural areas in Chile. Parasites Vectors 11, 1–7.
